# Chain-breaking antioxidant activity of reduced forms of mitochondria-targeted quinones, a novel type of geroprotectors

**DOI:** 10.18632/aging.100049

**Published:** 2009-05-12

**Authors:** Vitaly A. Roginsky, Vadim N. Tashlitsky, Vladimir P. Skulachev

**Affiliations:** ^1^N. Semenov Institute of Chemical Physics, Russian Academy of Sciences, 119991 Moscow, Russian Federation; ^2^Center of Mitoengineering, Moscow State University, 119991, Moscow, Russian Federation; ^3^Chemical Department, Moscow State University, 119991 Moscow, Russian Federation; ^4^A.N.Belozersky Institute of Physico-Chemical Biology, Moscow State University, 119991 Moscow, Russian Federation

**Keywords:** Geroprotector, mitochondria-targeted antioxidants, plastoquinone, ubiquinone, antioxidant activity, triphenylphosphonium

## Abstract

The chain-breaking antioxidant activities of reduced form of novel type of geroprotectors, mitochondria-targeted quinones (QH_2_) have quantitatively been measured for the first time. To this end, the chain peroxidation of methyl linoleate (ML) in Triton micelles was used as a kinetic testing model. The studied QH_2_ were lipophilic triphenylphosphonium cations conjugated by an aliphatic linker to an antioxidant, i.e. a ubiquinol moiety (MitoQH_2_) or plastoquinol moiety (SkQH_2_). The antioxidant activity was characterized by the rate constant k_1_ for the reaction between QH_2_ and the lipid peroxyl radical (LO_2_^·^) originated from ML: QH_2_ + LO_2_^·^ → HQ^·^ + LOOH. All the tested QH_2_ displayed a pronounced antioxidant activity. The oxidized forms of the same compounds did not inhibit ML peroxidation. The value of k_1_ for SkQH_2_ far exceeded k_1_ for MitoQH_2_. For the biologically active geroprotectors SkQ1H_2_, the k_1_ value found to be as high as 2.2 × 10^5^ M^-^^1^s^-^^1^, whereas for MitoQH_2_, it was 0.58 × 10^5^ M^-^^1^s^-^^1^. The kinetic behavior of QH_2_ suggested that SkQ1H_2_ can rather easily diffuse through lipid-water microheterogeneous systems.

## Introduction

**Figure 1.**
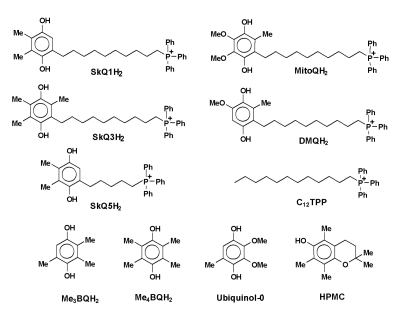
The structure of the mitochondria-targeted hydroquinones and other phenolics studied in this work.

The oxidative stress caused by reactive oxygen species (ROS) is assumed to significantly contribute to aging and numerous age-related pathologies. Mitochondria are known as a place, where the most intensive ROS production can occur. In the recent years, mitochondria-targeted antioxidants has been developed [1-4]. Research was the series of papers published by our group in 1969-1970, where mitochondria-addressed penetrating synthetic cations were described and the idea to use these cations as "electric locomotives" targeting non-charged compounds to mitochondria was put forward [5,6]. In the late nineties, Murphy and coworkers initiated the practical realization of this idea [1,7-9]. They synthesized and tested several mitochondria-targeted antioxidants conjugated to the lipophilic alkyltriphenylphosphonium cations. The ubiquinone moiety linked to triphenylphosphonium cation by C_10_ aliphatic chain, MitoQ (Figure 1), seemed to be the most promising [1,4,9].

In 2005, an attempt was undertaken in our group to replace the ubiquinone moiety in MitoQ by plasto-quinone. As a result, a series of mitochondria-targeted antioxidants named SkQ has been synthesized [2,10]. There were two main reasons for this modification [1]. Plastoquinone playing in chloroplasts the same role of an electron carrier as ubiquinone does in mitochondria always operates under conditions of oxidative stress (elevated oxygen concentration and an intensive ROS production) [2]. It was reported [11-13] that the reactivity of the "tailless" plastoquinol analogs to the peroxyl radicals was indeed higher than that of natural ubiquinols. The advantage of mitochondria-targeted quinones of SkQ type over MitoQ was recently demonstrated by using several biological models. In particular, it was found that very low doses of SkQ1 (nmol/kg per day) prolong life of podospora, ceriodaphnia, drosophila and mice. In mice, SkQ1 doubled median lifespan arrested development of such traits of the senescence process as involution of thymus and decline of other immunity mechanisms; osteoporosis; disappearance of regular estrous cycles in females, cataract, retinopathies, balding, catinies, hypothermia, chromosome aberrations, peroxidation of lipids and proteins, etc. [10,14-20].

Until recently, the reactivity of the mitochondria-targeted antioxidants has, in fact, not been quantitatively determined. This was done in the present paper. The structure of the compounds studied is presented in Figure 1. The chain-breaking antioxidant activity was characterized by the rate constant for reaction of QH_2_ with the lipid peroxyl radical, LO_2_^•^, formed from ML or cardiolipin: LO_2_^•^ + QH_2_¾→ LOOH + QH^•^ k_1_ [1] which competes with the reaction of chain propagation of lipid peroxidation LO_2_^•^ + LH (+O_2_) ¾→ LOOH + LO_2_^•^ k_2_[2].

## Results

**Figure 2.**
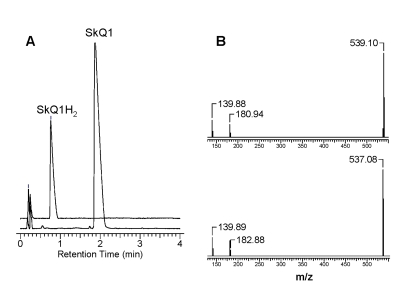
The reduction of SkQ1 by NaBH_4_ as studied by UPLC-MS-MS analysis. (**A**) - Reverse-phase HPLC chromatograms before and after the addition of NaBH_4_. (**B**) - MS/MS spectra of SkQ1 before reduction (at the bottom) and after reduction (at the top). Details of the protocol are given in the text.

Figure 2 shows that SkQ1 is almost completely reduced to SkQ1H_2_ by NaBH_4_. For SkQ1, the m/z value was found to be 537.08, which corresponds to the theoretically calculated one. As expected, the m/z value for SkQ1H_2_ proved to be 539.1, i.e. m/z increased by two units as compared with that for SkQ1. Similar results were also obtained for the reduction of other mitochondria-targeted quinones.

The non-inhibited oxidation of ML in Triton micelles is a chain process, which rate, R_0_, was found to be proportional to [ML] and square root of [AAPH] (not shown) as it was reported in our preceding papers [21,22]. Such relationships are also inherent in the lipid peroxidation in other aqueous microheterogeneous systems [23-25]. They correspond to the "classic" kinetic scheme with bimolecular chain termination [26,27].

**Figure 3.**
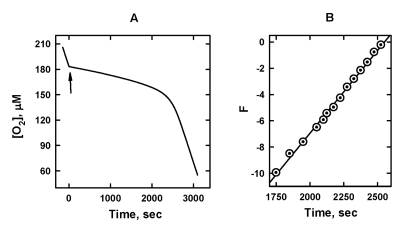
The effect of 5 μM SkQ1H_2_ on the kinetics of oxygen consumption caused by oxidation of 20 mM ML in micellar solution of 50 mM Triton X-100 in 50mM phosphate buffer, pH 7.4, 37 °C. Oxidation was initiated by 3 mM AAPH. (**A**) [O_2_] trace; arrow shows addition of SkQ1H_2_. (**B**) plot A in the axes of Eq. 7.

 AAPH + LH + (O_2_) ¾→ LO_2_^•^ + products R_IN_ (0) LO_2_^•^ + LH + ¾→ LOOH + L^•^ k_2_ [2] L^•^ + O_2_¾→ LO_2_^•^ k_3_ [3] LO_2_^•^ + LO_2_^•^¾→ products 2k_4_ [4] All the tested QH_2_ displayed a pronounced chain-breaking antioxidant activity as this is exemplified by Figure 3 for SkQ1H_2_. When SkQ1H_2_ was added, the rate of oxidation, R, dramatically decreased. As SkQ1H_2_ was progressively consumed due to reaction [1], R increased with time and eventually reaches the level of non-inhibited oxidation. As a result, the pronounced induction period was observed (Figure 3A).

**Figure 4.**
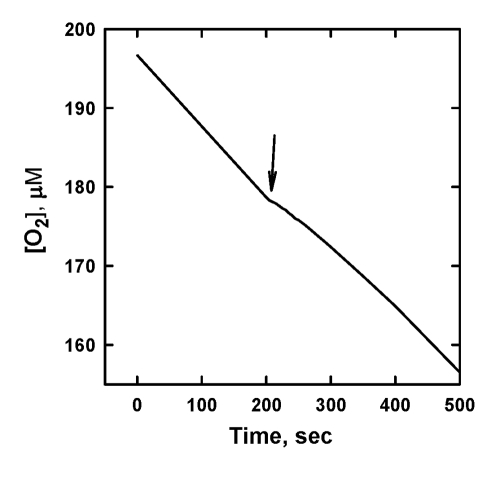
The effect of addition of 10 μM SkQ1 on the kinetics of oxygen consumption during the oxidation of 20 mM ML in 50 mM micellar solution of 50 mM Triton X-100 in 50mM phosphate buffer, pH 7.40, 37 °C, initiated by 3 mM AAPH. Arrow shows the moment when SkQ1 was added.

Quantitatively similar [O_2_] traces were observed with all the other tested QH_2_ as well as with α-tocopherol and its synthetic analog 6-hydroxy-2,2,5,7,8-pentamethylchromane (HPMC). As for C_12_TPP, a compound that has no hydroquinone moiety (Figure 1), it did not display any inhibiting activity (not shown). Meanwhile, oxidized form of SkQ1 showed a weak inhibition of ML oxidation, but only during a very short period of time (Figure 4). Most likely, the inhibition is caused in this case by a minor contamination of SkQ1H_2_ to SkQ1. A similar effect was also observed with other mitochondria-targeted Q. This suggests that mitochondria-targeted quinones by themselves do not act as a chain-breaking antioxidant.

The reduced forms of mitochondria-targeted quinones studied in this work are p-hydroquinones. Acting as chain-breaking antioxidants during the chain peroxidation of styrene p-hydroquinones, "tailless" analogs of mitochondria-targeted antioxidants show a very high inhibiting activity [11], sometimes comparable with that of α-tocopherol (k_1_ = 3.3 × 10^6^M^-^^1^s^-^^1^ [26]). For instance, k_1_ for Me_3_BQH_2_ was found to be as much as 2.2 × 10^6^M^-^^1^s^-^^1^ (Table 1). The behavior of p-hydroquinones in such a system does not differ from that of monophenolic antioxidants [26,27]. The situation dramatically changes when going to the peroxidation of ML in aqueous micelles [12,28]. The matter is that p-hydroxy-substituted phenoxyl radicals QH^•^ formed in reaction [1] having, as a rule, pK less than 5 [29] undergo fast deprotonation at neutral pH: QH^•^¾→ Q^•^^-^ + H^+^ [5] with the formation of semiquinone anion, Q^•^^-^, which reacts readily with molecular oxygen, forming O_2_^•^^-^ [30,31]: Q^•^^-^ + O_2_¾→ Q + O_2_^•^^-^ [6] In turn, O_2_^•^^-^ may react with oxidation substrate and QH_2_, most likely in its protonated form, HO_2_^•^. Both reactions result in a decrease in the inhibitory activity of QH_2_ [28]. SOD removes O_2_^•^^-^and thus arrests the mentioned undesirable reactions with the participation of O_2_^•^^-^ (HO_2_^•^). This was a reason why SOD was always added to our system.

**Table 1.** Kinetic parameters characterizing the antioxidant activity of the reduced forms of mitochondria-targeted quinones and their analogs in micellar solution of 50 mM Triton X-100, 50 mM phosphate buffer, pH 7.4, at 37 °C. Oxidation of ML or cardiolipin was initiated by AAPH. Notes: nd - not determined; ^a^ structures of QH2 are given in Figure 1; ^b^ figures in brackets are the number of independent experiments; ^c^ ML is replaced by cardiolipin; ^d^ determined during styrene oxidation in the bulk.

QH_2_^a^	k_1_/k_2_^b^	k_1 _× 10^5^, M^-^^1^s^-^^1^
SkQ1H_2_	3670 ± 280 (7) 1980 ± 170 (3)^c^	2.2 ± 0.2 nd
SkQ3H_2_	2720 ± 210 (4)	1.6 ± 0.1
SkQ5H_2_	2670 ± 180 (5)	1.6 ± 0.1
MitoQH_2_	970 ± 55 (6) 520 ± 37 (3)^c^	0.58 ± 0.03 nd
DMQH_2_	1260 ± 85 (4)	0.76 ± 0.5
Me_3_BQH_2_	2170 ± 130 (4)	1.3 ± 0.1 23^d^
Me_4_BQH_2_	5020 ± 380 (3)	3.0 ± 0.2
Ubiquinol-0	700 ± 45 (3)	0.42 ± 0.03 4.4^d^
α-tocopherol	1170 ± 70 (4)	0.70 ± 0.04
HPMC	8680 ± 700 (4)	5.2 ± 0.4

The [O_2_] traces recorded during the induction period of the inhibited oxidation of ML were used to determine k_1_. On the base of a reductive kinetic scheme, which includes reactions (0), [1], [2], and [4], the following equation can be deduced [11,12] where [LH] is the concentration of the oxidation substrate (in our case ML). Figure 3B depicts the original [O_2_] trace (Figure 3A) in the axes of Eq. [7]. It is seen that the plot of F vs. time is a straight line as predicted by Eq. [7]. The kinetic behavior of all the other QH_2_ studied proved to be was similar. The value of k_1_/k_2_ can be calculated from the slope of this straight line by using Eq. 7. It should be noted that this way of calculation of k_1_/k_2_ does not require the knowledge in R_IN_ and the starting concentration of QH_2_. The values of k_1_/k_2_ are listed in Table 1. The absolute values of k_1_were calculated from k_1_/k_2_ assuming k_2_ = 60 M^-^^1^s^-^^1^ [22].

The k_1_ values are also listed in Table 1.

**Figure 5.**
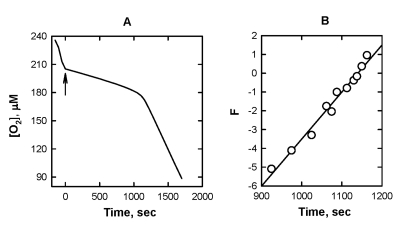
The effect of addition of 10 μM SkQ1H_2_ on the kinetics of oxygen consumption during the oxidation of 2.6 mg mL^-^^1^ cardiolipin 50 mM micellar solution of 50 mM Triton X-100 in 50mM phosphate buffer, pH 7.40, 37 °C, initiated by 3 mM AAPH. Arrow shows the moment when SkQ1H_2_ was added.

With two QH_2_, SkQ1H_2_ and MitoQH_2_, similar experiments were conducted by using the same testing system, but with substituting ML by cardiolipin, the most oxidizable phospholipid component in mitochondria membranes [32,33]. As seen from Figure 5, both [O_2_] traces during the induction period of the inhibited oxidation and the plots of F vs. time are very similar to those for ML. The value of k_1_/k_2_ was calculated from the slope of the plot B (Figure 5) by using Eq. [7] assuming that each molecule of cardiolipin contains four fatty acid residue with 87 % linoleate in the cardiolipin sample used in this work (see http://www.avantilipids.com). These data are also presented in Table 1. Unfortunately, the absolute values of k_1_ could not be calculated, as k_2_ for the oxidation of cardiolipin has never been reported.

## Discussion

In this paper, the reactivity of the reduced forms of the mitochondria-targeted quinones as chain-breaking anti-oxidants has systematically been studied. As may be seen from Table 1, the k_1_ value for SkQ1H_2_, SkQ3H_2_ and SkQ5H_2_ are significantly higher than that for MitoQH_2_. This is in line with the data for simple "tailless" analogs of SkQ1H_2_ and MitoQH_2_, namely Me_3_BQH_2_, Me_4_BQH_2_ and Ubiquinol-0. The same tendency was earlier observed when effects of "tailless" analogues on the chain oxidation of styrene in bulk [11] and ML peroxidation in SDS micelles were studied [12]. Possible reasons why methyl-substituted p-hydroquinones are better antioxidants than methoxy-substituted p-hydroquinones were described elsewhere [11,26]. In brief, the effect under consideration is, the most probably, stereoelectronic by its nature. The matter is that o-methoxy group forms H-bond with oxygen belonging to the adjacent OH group. This causes the decrease in overlap between p-type orbital of oxygen atom of OH-group and the aromatic π-electron cloud (the increase of the dihedral angle between the aromatic ring and O - H bond). The latter results in strengthening O - H bond as compared with that in o-methyl substituted QH_2_, where such an intramolecular H-bond is absent.

Among mitochondria-targeted QH_2_ studied in this work, SkQ1H_2_ showed the highest reactivity towards the lipid peroxyl radicals (Table 1). This observation is in line with data obtained in our group by using several biological models [2,10,14]. However, we recognize that the highest value of k_1_ for SkQ1H_2_ is likely not the only reason for the outstanding biological activity of SkQ1. It should be taken into account that k_1_ given in Table 1 are effective values and cannot be directly attributed to the elementary reaction [1]. The genuine values of k_1_ can be determined during the chain oxidation in non-polar media, for instance in styrene [11,34,35]. When going to the oxidation of fatty acid (ester) in bulk [12,36]and further to the oxidation in aqueous micelles and liposomes [12,26,37], the experimentally determined k_1_ values significantly decrease, nearly by one order of magnitude (see data for ubiquinol-0, Table 1). A reason for such a reduction of k_1_ was repeatedly discussed. The mentioned decrease in k_1_ is not specific of QH_2_. A similar effect has earlier been also reported for the oxidation inhibited by monophenolics [25,26,37,38]. The formation of H-bonds between the OH-group of phenolics and the carboxy-group of ML has been suggested as the main reason for the k_1_ decrease when going from the oxidation of non-polar hydrocarbon to that of fatty acid (ester) [36]. Recently, hydrogen bonding between phenols and fatty acid esters was directly observed by using the NMR technique [39]. Most likely, this is also true for QH_2_ studied in this work. The further decrease in k_1_ when going from ML oxidation in bulk to that in aqueous micelles may be explained by the additional formation of H-bonds between QH_2_ and water molecules as this was earlier suggested for monophenolics [23,37,38].

A general specific feature of reduced forms of the studied mitochondria-targeted quinoles is that their reactivity is actually very close to that of their "tailless" analogs (Table 1). This is in contrast to the couple "α-tocopherol having the long aliphatic chain its "tailless" analog HPMC. The k_1_ value for α-tocopherol is nearly one order of magnitude lower than that for HPMC (Table 1). This effect was reported to be even more pronounced in the SDS micelles [23,37,38]. The essential feature of our testing system and related microheterogeneous systems is that the concentration of the antioxidants tested is much lower than that of the oxidation substrate (in our case ML). While every micelle (microreactor) contains several molecules of ML, only a few micelles contain an antioxidant. Under these conditions, a fast LO_2_^•^ reduction by an antioxidant is possible only if an antioxidant is capable of fast transferring from one microreactor to another, the characteristic time of this transfer being shorter than the time of the occurrence of a single kinetic chain. The antioxidants with a rather long aliphatic residue like α-tocopherol commonly do not meet such a requirement [37]. The fact that the values of k_1_ for the mitochondria-targeted quinols actually do not differ from that of their "tailless" analogs (Table 1) means that all of them are capable of the fast transfer from one microreactor to another. This is in line with a high reported ability of SkQ and MitoQ to easily penetrate through biological membranes [14].

## Materials and methods

Methyl linoleate and Triton X-100 were purchased from Sigma, heart bovine cardiolipin disodium salt was received from Avanti PolarLipids. The water-soluble initiator 2,2'-azobis(2-amidinopropan) dihydrochloride (AAPH) was obtained from Polysciences. NaH_2_PO_4_ and Na_2_HPO_4_ of the highest quality used to prepare buffer solutions were purchased from Merck. The mitochondria-targeted quinones, SkQ1, SkQ3, SkQ5, MitoQ, DMQ as well as C_12_TPP (see Figure 1) were synthesized in the Mitoengineering Centre of Moscow State University [2]. Trimethylhydroquinone (Me_3_BQH_2_) was purchased from Aldrich; 2,3-dimethoxy-5-methyl-benzoqyuinone (ubiquinone-0) was from Sigma; tetramethylbenzoquinone (Me_4_BQ) was from EGA Chemie. All the other chemicals were of highest available quality.

The reduced forms of the mitochondria-targeted quinones (QH_2_) were produced by the reduction of corresponding quinones by NaBH_4_ in the mixture of 50 mM NaH_2_PO_4_ (pH 5.0) with ethanol. This process was under control of UPLC-MS-MS (see below). Reduced forms of ubiquinone-0 and tetramethylhydroquinone (Me_4_BQH_2_) were produced by reduction of the quinones by Zn powder [21]. The buffer solution (pH 7.40 ± 0.02) was prepared by mixing 50 mM solutions of NaH_2_PO_4_ and Na_2_HPO_4_. In turn, the solutions of the individual sodium phosphates were prepared with doubly distilled water and were purged from traces of transition metals by Chelex-100 resin (Bio-Rad).

HPLC-diode array detection-electrospray ionization tandem mass spectrometry analysis (UPLC-MS-MS) was performed using an ACQUITY system (Waters, Milford, MA, USA). Chromatography was carried out using an ACQUITY BEH C18 column (2.1 x 50 mm, 1.7 μm) eluted with a gradient of 40-60% acetonitrile (4 min) and 20 mM acetic acid (pH 3.0) delivered at a flow rate of 0.5 mL per min. UV-monitoring was performed at 280 mm. An injection volume of 11.2 μL (full loop) was used in all cases. A Quattro triple-quadrupole mass spectrometer (Micromass-Waters) fitted with a Z-Spray ion interface was used for analyses. Ionization was achieved using electrospray in a positive ionization mode. The following conditions were found to be optimal for the analysis of SkQ1: capillary voltage, 3.0 kV; source block temperature, 120°C; and desolvatation gas (nitrogen) heated to 450°C and delivered at a flow rate of 800 L h^-^^1^; cone voltage, 55 V; cone Gas Flow rate, 50 L h^-^^1^. MassLynx 4.0 software (Waters) was used for processing.

The standard testing system was composed of 50 mM buffer, pH 7.4, 50 mM Triton X-100, 2-4 mM AAPH, 8-20 mM ML and 20 unit mL^-^^1^ SOD. In some experiments, ML was replaced by cardiolipin. The kinetics of oxygen consumption accompanied ML (cardiolipin) oxidation were studied with a computerized 5300 Biological Oxygen Monitor (Yellow Springs Instruments Co., USA) with a Clark electrode as a sensor. The rate of oxidation was measured as a slope of [O_2_] traces. Experiments were conducted at 37.0 ± 0.1 °C. ML was added to preliminarily thermostated micellar solution of Triton X-100 and AAPH in buffer. Monitoring was started 3-5 min after ML addition and the rate of non-inhibited oxidation (R_0_) was measured. The tested compounds were then added to a reaction chamber under steady monitoring as a stock solution by using a Hamilton micro-syringe. In more detail, the protocol was described elsewhere [12,21,22].
